# Heavy Metals and Essential Metals Are Associated with Cerebrospinal Fluid Biomarkers of Alzheimer’s Disease

**DOI:** 10.3390/ijms24010467

**Published:** 2022-12-27

**Authors:** Mirjana Babić Leko, Matej Mihelčić, Jasna Jurasović, Matea Nikolac Perković, Ena Španić, Ankica Sekovanić, Tatjana Orct, Klara Zubčić, Lea Langer Horvat, Nikolina Pleić, Spomenka Kiđemet-Piskač, Željka Vogrinc, Nela Pivac, Andrea Diana, Fran Borovečki, Patrick R. Hof, Goran Šimić

**Affiliations:** 1 Department of Neuroscience, Croatian Institute for Brain Research, University of Zagreb School of Medicine, 10000 Zagreb, Croatia; 2 Department of Medical Biology, University of Split School of Medicine, 21000 Split, Croatia; 3 Department of Mathematics, University of Zagreb Faculty of Science, 10000 Zagreb, Croatia; 4 Analytical Toxicology and Mineral Metabolism Unit, Institute for Medical Research and Occupational Health, 10000 Zagreb, Croatia; 5 Ruđer Bošković Institute, Division of Molecular Medicine, 10000 Zagreb, Croatia; 6 Department of Neurology, General Hospital Varaždin, 42000 Varaždin, Croatia; 7 Laboratory for Neurobiochemistry, Department of Laboratory Diagnostics, University Hospital Centre Zagreb, 10000 Zagreb, Croatia; 8 Laboratory of Neurogenesis and Neuropoiesis, Department of Biomedical Sciences, University of Cagliari, Monserrato, 09042 Cagliari, Italy; 9 Department for Functional Genomics, Center for Translational and Clinical Research, University of Zagreb Medical School, University Hospital Center Zagreb, 10000 Zagreb, Croatia; 10 Nash Family Department of Neuroscience, Friedman Brain Institute, Ronald M. Loeb Center for Alzheimer’s Disease, Icahn School of Medicine at Mount Sinai, New York, NY 10029, USA

**Keywords:** Alzheimer’s disease, heavy metals, essential metals, cerebrospinal fluid, biomarker, arsenic, mercury, cadmium, iron, zinc, calcium

## Abstract

Various metals have been associated with the pathogenesis of Alzheimer’s disease (AD), principally heavy metals that are environmental pollutants (such as As, Cd, Hg, and Pb) and essential metals whose homeostasis is disturbed in AD (such as Cu, Fe, and Zn). Although there is evidence of the involvement of these metals in AD, further research is needed on their mechanisms of toxicity. To further assess the involvement of heavy and essential metals in AD pathogenesis, we compared cerebrospinal fluid (CSF) AD biomarkers to macro- and microelements measured in CSF and plasma. We tested if macro- and microelements’ concentrations (heavy metals (As, Cd, Hg, Ni, Pb, and Tl), essential metals (Na, Mg, K, Ca, Fe, Co, Mn, Cu, Zn, and Mo), essential non-metals (B, P, S, and Se), and other non-essential metals (Al, Ba, Li, and Sr)) are associated with CSF AD biomarkers that reflect pathological changes in the AD brain (amyloid β_1–42_, total tau, phosphorylated tau isoforms, NFL, S100B, VILIP-1, YKL-40, PAPP-A, and albumin). We used inductively coupled plasma mass spectroscopy (ICP-MS) to determine macro- and microelements in CSF and plasma, and enzyme-linked immunosorbent assays (ELISA) to determine protein biomarkers of AD in CSF. This study included 193 participants (124 with AD, 50 with mild cognitive impairment, and 19 healthy controls). Simple correlation, as well as machine learning algorithms (redescription mining and principal component analysis (PCA)), demonstrated that levels of heavy metals (As, Cd, Hg, Ni, Pb, and Tl), essential metals (Ca, Co, Cu, Fe, Mg, Mn, Mo, Na, K, and Zn), and essential non-metals (P, S, and Se) are positively associated with CSF phosphorylated tau isoforms, VILIP-1, S100B, NFL, and YKL-40 in AD.

## 1. Introduction

Macroelements are those elements that the body needs more than any other mineral and include sodium (Na), potassium (K), calcium (Ca), magnesium (Mg), chlorine (Cl), phosphorus (P), and sulfur (S). Microelements are those elements that are required for good health in very small amounts, such as chromium (Cr), copper (Cu), fluorine (F), iodine (I), iron (Fe), manganese (Mn), molybdenum (Mo), selenium (Se), zinc (Zn), and cobalt (Co). Some of these microelements are also metals and are regarded as essential for human health in trace amounts, for example, Fe, Zn, Cu, Mn, Cr, Mo, Se, and Co (Co because it is necessary for the formation of vitamin B12–cobalamin). However, non-essential metals are considered harmful to human health and the environment. These include four heavy metals: arsenic (As), cadmium (Cd), lead (Pb), and mercury (Hg) that can cause neurodegenerative changes [1] and have been also associated with the development of Alzheimer’s disease (AD) [2]. People are exposed to heavy metals through water, soil, and air. Heavy metals can cross the blood–brain barrier (BBB), accumulate in the brain, and bypass BBB, entering the brain directly through the olfactory pathway [3].

There is evidence that essential metal homeostasis is disturbed in the brain of AD patients [4,5,6]. Here, in the first place, we mean metals that are present in the body under normal circumstances and are essential for the functioning of numerous enzymes. Calcium, Na, and Mg are the most abundant metals in the human body, while metals such as Cu, Fe, Zn, Cr, Mo, Mn, and Co are found only in traces. Metal ions stabilize proteins and nucleic acids are crucial for the function of metalloenzymes located in the active site of enzymes and act as secondary messengers. 

An increase in the concentration of heavy metals and altered homeostasis of essential metals is observed in AD brains, which contributes to tau protein hyperphosphorylation [7,8] and Aβ aggregation [9,10]. Additionally, it was shown that essential metals such as Fe, Zn, and Cu accumulate within senile plaques and promote Aβ and tau pathology. Increased levels of metals can also contribute to the impairment of the BBB [11], oxidative stress [12], altered calcium homeostasis [13], neuroinflammation [14], apoptosis, and necrosis of neurons [15,16]. Various studies measured both heavy metals and essential metals in plasma and cerebrospinal fluid (CSF) of patients with dementia (reviewed in [17]), but due to high variability among studies, metals are still not used as biomarkers in the diagnostics of AD. However, various metal chelators were tested as potential therapeutic agents in AD (reviewed in [18]), clioquinol in particular, whose action is based on the removal of excess metal ions in the brain. This compound showed good results in the second phase of clinical trials [19], but due to manufacturing difficulties, it did not progress further [20]. PBT2, a homolog of clioquinol, showed better therapeutic effects, but its ability to reduce the pathological changes associated with the accumulation of Aβ in the brain of AD patients could not be demonstrated (http://www.alzforum.org/news/research-news/pbt2-takes-dive-phase-2-alzheimers-trial (accessed on 20 November 2022)). Clioquinol and PBT2 remove excess copper and zinc by acting as ionophores, and clioquinol probably also removes iron by chelation [4,21]. Other iron chelators such as epigallocatechin-3-gallate and M-30 have also shown beneficial effects on pathological changes characteristic of AD *in vivo* and *in vitro*, and deferoxamine has also been tested on patients (reviewed in [20]).

The main goal of this study was to test the association of various CSF biomarkers of AD with macro- and microelements measured in CSF and the plasma of AD patients, patients with mild cognitive impairment (MCI), and healthy controls (HC). We assessed such associations with AD-related pathological changes reflected by the levels of eleven AD biomarkers in CSF, whose association with AD is established. 

## 2. Results

### 2.1. Correlation

**Figure 1:**
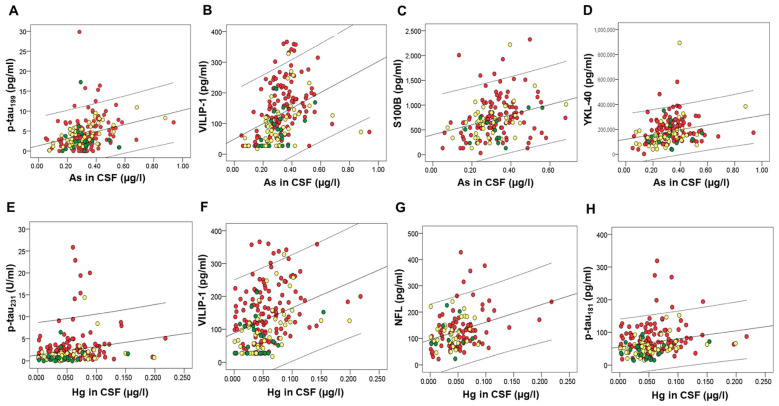
Correlation of CSF biomarkers of AD with As (**A**–**D**) and Hg (**E**–**H**) measured in CSF. Red circles represent AD patients, yellow circles represent MCI patients, while green circles represent healthy controls.

**Figure 2:**
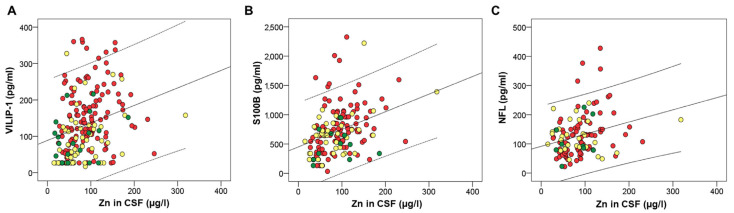
Correlation of CSF biomarkers VILIP-1 (**A**), S100B (**B**), and NFL (**C**) of AD with Zn measured in CSF. Red circles represent AD patients, yellow circles represent MCI patients, while green circles represent healthy controls.

**Figure 3:**
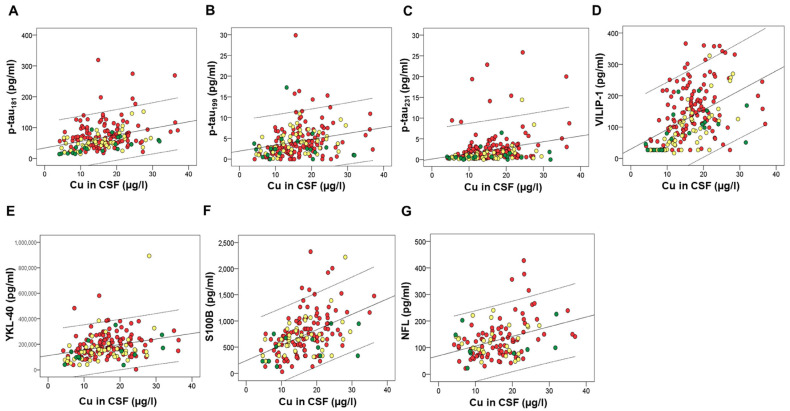
Correlation of CSF biomarkers of AD – p-tau_181_ (**A**), ptau_199_ (**B**), ptau_231_ (**C**), VILIP-1 (**D**), YKL-40 (**E**), S100B (**F**), and NFL (**G**) with Cu measured in CSF. Red circles represent AD patients, yellow circles represent MCI patients, while green circles represent healthy controls.

**Figure 4:**
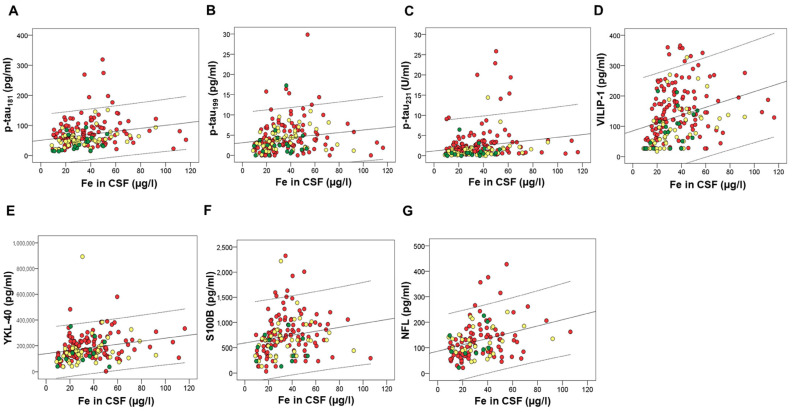
Correlation of CSF biomarkers of AD – p-tau_181_ (**A**), ptau_199_ (**B**), ptau_231_ (**C**), VILIP-1 (**D**), YKL-40 (**E**), S100B (**F**), and NFL (**G**) with Fe measured in CSF. Red circles represent AD patients, yellow circles represent MCI patients, while green circles represent healthy controls.

Several macro- and microelements correlated with CSF AD biomarkers. However, after applying Bonferroni correction for multiple comparisons, we considered statistically significant only those correlations with *p*-values ≤ 0.001. These correlations are presented in Table 1. Macro- and microelements that correlated with the high number of CSF AD biomarkers were all measured in CSF; As and Hg (Figure 1), Zn (Figure 2), Cu (Figure 3), Fe (Figure 4), S, K, Se, Co, Mn, Ni, Na, Mg, Tl, and Li (Table 1).

### 2.2. Principal Component Analysis

Principal component analysis (PCA) showed differences in all macro- and microelements measured in CSF (except for P, which was measured in the relatively small number of subjects) and plasma. Factor analysis of CSF macro- and microelements included 23 parameters that were classified into 6 groups (factors) after analysis (Table 2). Factors explained 73.2% of the total variance in analyzed CSF macro- and microelements (with Bartlett’s test of sphericity of p < 0.001 and Kaiser–Meyer–Olkin measure of sampling adequacy of 0.846). Factor analysis of plasma macro- and microelements included 21 parameters that were classified into six groups (factors) after analysis (Table 3). Factors explained 66.1% of the total variance in analyzed plasma macro- and microelements (with Bartlett’s test of sphericity of p < 0.001 and Kaiser–Meyer–Olkin measure of sampling adequacy of 0.781).

**Figure 5:**
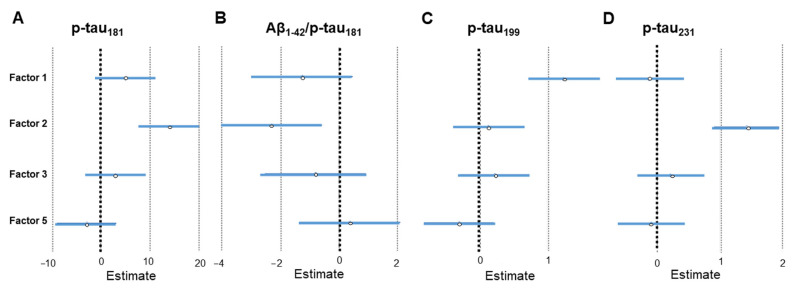
Association of CSF AD biomarkers – p-tau_181_ (**A**), Aβ_1–42_/p-tau_181_ ratio (**B**), p-tau_199_ (**C**), and p-tau_231_ (**D**) with CSF macro- and microelement groups revealed by PCA. Effect sizes and 95% confidence intervals are given by linear regression analysis (statistically significant factors are considered those represented with horizontal lines that do not intersect with the vertical line at 0). Factor 1; CSF As, Ba, Ca, Co, Cu, Fe, K, Mg, Mn, Na, Ni, S, Se, Sr, Tl, and Zn; Factor 2; CSF Al, Cd, and Pb; Factor 3; CSF B and Li; Factor 5; CSF Hg and Mo.

**Figure 6:**
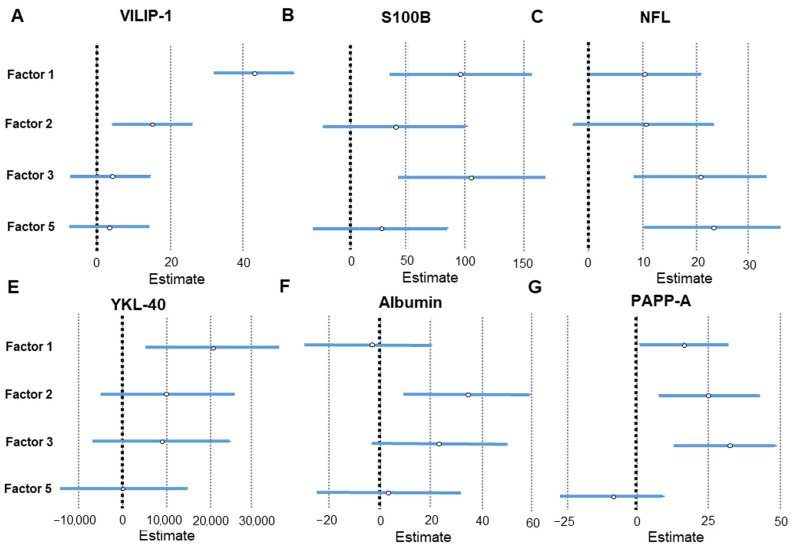
Association of CSF AD biomarkers – VILIP-1 (**A**), S100B (**B**), NFL (**C**), YKL-40 (**E**), albumin (**F**), and PAPP-A (**G**) with CSF macro- and microelement groups revealed by PCA. Effect sizes and 95% confidence intervals are given by linear regression analysis (statistically significant factors are considered those represented with horizontal lines that do not intersect with the vertical line at 0). Factor 1; CSF As, Ba, Ca, Co, Cu, Fe, K, Mg, Mn, Na, Ni, S, Se, Sr, Tl, and Zn; Factor 2; CSF Al, Cd, and Pb; Factor 3; CSF B and Li; Factor 5; CSF Hg and Mo.

**Figure 7:**
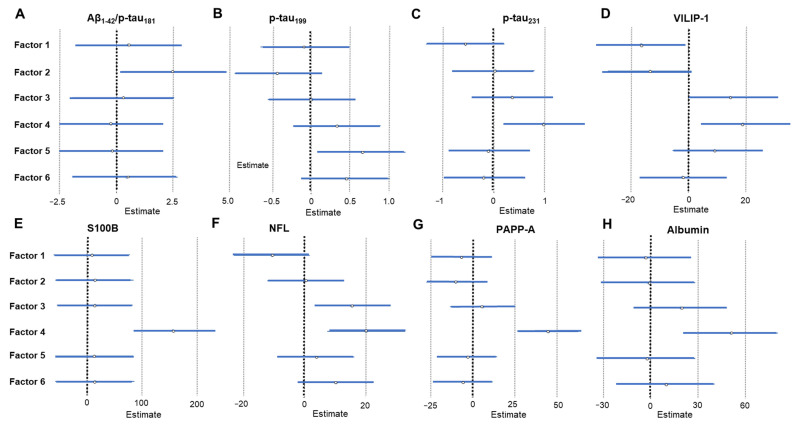
Association of CSF AD biomarkers – Aβ_1–42_/p-tau_181_ ratio (**A**), p-tau_199_ (**B**), p-tau_231_ (**C**), VILIP-1 (**D**), S100B (**E**), NFL (**F**), PAPP-A (**G**), and albumin (**H**) with plasma macro- and microelement groups revealed by the PCA. Effect sizes and 95% confidence intervals are given by linear regression analysis (statistically significant factors are considered those represented with horizontal lines that do not intersect with the vertical line at 0). Factor 1; plasma Ca, Co, Cu, Mg, Mn, Na, P, S, Se, Tl, and Zn; Factor 2; plasma B, Cd, Li, Mo, and Pb; Factor 3; plasma As and Hg; Factor 4; plasma Ni; Factor 5; plasma Sr; Factor 6; plasma Fe.

The results of linear regression analysis that compared the factors obtained by PCA analysis of CSF macro- and microelements with CSF AD biomarkers are given in Table 4 and Figure 5 and Figure 6. The results of linear regression analysis that compared the factors obtained by PCA of plasma macro- and microelements with CSF AD biomarkers are given in Table 5 and Figure 7. The most interesting finding is that following PCA heavy metals are distinctly grouped (Cd, Pb, and Al in CSF; As and Hg in plasma). The metals measured in CSF (Cd, Pb, and Al) associated positively with tau phosphorylated at Thr 181 (p-tau_181_), Thr 231 (p-tau_231_), visinin-like protein 1 (VILIP-1), pregnancy-associated plasma protein A, pappalysin-1 (PAPP-A) and albumin, and negatively with the Aβ_1–42_/p-tau_181_ ratio. The metals measured in plasma (As and Hg) showed a positive association with VILIP-1 and neurofilament light chain (NFL) levels. PCA also showed that Ni measured in plasma was positively associated with various CSF AD biomarkers. In CSF, B and Li grouped together (Factor 3) and showed a positive association with NFL, S100 calcium-binding protein B (S100B), and PAPP-A.

### 2.3. Redescription Mining

Redescription mining showed an association of CSF AD biomarkers with macro- and microelements measured in CSF (Table 6), and plasma (Table 7), as well as an association of CSF AD biomarkers with macro- and microelements measured both in CSF and in plasma (Table 7). Information on age, Mini-Mental State Examination (MMSE), and *APOE* (apolipoprotein E) genotype were included in the analysis. Redescription mining gave us in total of 2648 redescriptions. Redescriptions that depict more closely: (1) AD patients, (2) both AD and MCI patients, (3) MCI patients, (4) MCI patients and HC, and (5) HC were extracted (Table 6, Table 7). When analyzing the association of CSF AD biomarkers with macro- and microelements measured in CSF, VILIP-1 occurred together with Se in 81 redescriptions, and Ba and Cu in 69 redescriptions. P-tau_181_ occurred together with Se in 67 redescriptions and Cu in 59 redescriptions, while chitinase-3-like protein 1 (YKL-40) was associated with Se and Cu in 52 and 41 redescriptions, respectively. When analyzing the association of CSF AD biomarkers with macro- and microelements measured in plasma, PAPP-A occurred together with the following elements (the number of redescriptions is shown in parentheses): Li (281), Ca (279), Na (192), P (191), Ni (177), Hg (172), Se (164), Sr (130), and Mo (125) (only the most significant associations are presented). Total tau (t-tau) occurred together with Ca (181), Li (166), P (126), and Na (125); YKL-40 with Ca (173), Li (171), P (129), and Na (118); p-tau_231_ with Li (168), Ca (159), P (122), and Se (116); and p-tau_181_ with Li (122), and Ca (125).

## 3. Discussion

In this study, we used three different statistical methods to test the association of macro- and microelements with CSF biomarkers of AD. All three methods (simple correlation, redescription mining, and PCA) demonstrated some association of macro- and microelements with CSF AD biomarkers. Macro- and microelements that positively correlated with a high number of CSF AD biomarkers were As, Hg, Zn, Cu, Fe, S, K, Se, Co, Mn, Ni, Na, Mg, Tl, and Li (all elements measured in CSF). PCA further confirmed the association of these macro- and microelements with CSF AD biomarkers. Following PCA, heavy metals are distinctly grouped (Cd, Pb, and Al in CSF; As and Hg in plasma). The metals measured in CSF (Cd, Pb, and Al) associated positively with p-tau_181_, p-tau_231_, VILIP-1, PAPP-A, and albumin, and negatively with the Aβ_1–42_/p-tau_181_ ratio. The metals measured in plasma (As and Hg) showed a positive association with VILIP-1 and NFL levels. PCA also showed that Ni measured in plasma was positively associated with various CSF AD biomarkers. The redescription mining algorithm successfully clustered individuals by the combination of CSF AD biomarkers with macro- and microelements measured in CSF, CSF AD biomarkers with macro- and microelements measured in plasma, and CSF AD biomarkers with macro- and microelements measured both in CSF and in plasma. Using this algorithm, we extracted those redescriptions that depict more closely: AD patients, both AD and MCI patients, MCI patients, MCI patients and HC, and HC. Additionally, redescription mining showed the association of Ca, Li, P, and Na measured in plasma with various CSF AD biomarkers.

Several studies investigated the association of heavy metals, essential and non-essential metals, and essential non-metals with CSF AD biomarkers. Hock et al. showed a positive correlation between Hg blood levels and CSF amyloid β_1–42_ (Aβ_1–42_) levels [23]. A study on 28 AD patients and 25 HC found a negative correlation between serum Cu levels with CSF Aβ_1–42_, and a positive correlation with CSF t-tau levels [24], while oral intake of Cu showed no effect on CSF Aβ_1–42_, t-tau and p-tau levels in 68 AD patients [25]. Strozyk et al. observed a negative correlation between CSF Cu, Zn, Fe, Mn, and Cr levels and CSF Aβ_1–42_ levels [26]. A recent study that included 20 AD patients, 10 HC, and 10 patients with cerebral amyloid angiopathy (CAA) observed a negative correlation between CSF Fe levels and CSF Aβ_1–42_ levels (with Ni, Cr, Zn, Mn, Co, Cu, Aβ_1–40_, t-tau, p-tau_181_, and NFL being also measured in CSF) [27]. Kushnir et al. did not observe an association between CSF Ca levels and CSF Aβ_1–42_, t-tau, and p-tau [28], while Ma et al. observed a negative correlation between serum Ca levels and CSF Aβ_1–42_ levels and no association with CSF t-tau and p-tau_181_ (811 MCI patients and 413 HC; [29]). Blood Se levels were not associated with plasma Aβ_1–42_ and t-tau levels [30], while CSF Se levels were negatively associated with CSF Aβ_1–42_, and showed no association with CSF t-tau and p-tau levels [31]. CSF Mn levels positively correlated with CSF t-tau and p-tau_181_, while CSF Cs levels correlated negatively with t-tau and p-tau_181_ levels and positively with CSF Aβ_1–42_ levels [32]. Blood Mn levels positively correlated with plasma Aβ_1–42_ and Aβ_1–40_ levels [33], while serum Mn negatively correlated with serum t-tau levels [34]. Mielke et al. reported that low serum K levels in mid-life are associated with low CSF Aβ_1–42_ levels later in life [35]. Shams et al. observed a positive association between CSF Fe and Cu levels with CSF Aβ_1–42_, t-tau, p-tau_181_, and CSF/serum albumin ratio, and a positive association between CSF Zn levels and CSF/serum albumin ratio [36].

Additionally, it was shown that VILIP-1 is a neuronal calcium sensor protein that contains an EF-hand structural domain. This domain can bind metal ions [37], such as Ca, Mg [38,39], Cd [40], and Zn [41]. S100B is a calcium-binding protein that also contains EF-hand and can bind Ca, Zn [42], Cd [43], Mg, and K [44]. A study in Atlantic sharpnose sharks (*Rhizoprionodon terraenovae*) showed that brain Hg levels positively correlated with CSF S100B levels [45], while rats prenatally exposed to Hg had a significant increase in S100B expression (the effect was reversed with Zn treatment; [46]). Studies in humans showed that children with acute Hg intoxication had significantly increased serum S100B levels [47], while individuals chronically exposed to Hg had an increase in mRNA S100B expression [48]. Levels of As, Pb, and Cd measured in the blood of the patients with multiple sclerosis positively correlated with serum S100B levels [49]. A study in mice showed that arsenic exposure causes an increase in serum S100B levels [50], while manganese exposure increases the expression of S100B in the brain [51]. Additionally, treatment with magnesium sulfate in patients with aneurysmal subarachnoid hemorrhage did not affect serum S100B levels [52] while in patients with eclampsia [53] and neonatal hypoxic-ischemic encephalopathy [54], this treatment lead to the decrease in CSF and serum S100B levels, respectively. Regarding the association of YKL-40 with metals, we found only one study in patients with bipolar disorder that showed no association between serum Zn and serum YKL-40 levels [55]. Regarding NFL, studies in experimental animals showed that As treatment leads to NFL disappearance [56], while Al treatment reduced NFL mRNA levels [57].

All phosphorylated tau isoforms showed a strong positive correlation with Se, while p-tau_181_ strongly correlated with Cu, p-tau_199_ with As, and p-tau_231_ with Co measured in CSF. Vinceti et al. also reported a positive association of CSF Se with CSF p-tau_181_ levels [58]. However, the majority of the studies associated Se deficiency with increased risk of AD [59,60,61,62,63], with proposed Se supplementation as valuable in AD treatment [64]. Previous studies showed that both Cu [65,66] and As [7,67,68] induce tau phosphorylation, while exposure to Co induces age-dependent neurodegeneration in mice [69]. The neurodegeneration marker VILIP-1 showed the strongest correlation with CSF Se and Na. Se exerts its biological effects mainly through selenoproteins [70]. Similarly to VILIP-1 [71], selenoproteins are involved in the regulation of calcium homeostasis [72], and as such the strong correlation between VILIP-1 and Se observed in this study is not surprising. Previous studies associated increased Na levels with AD [5,73,74,75]. Another marker of neurodegeneration (NFL) and markers of glial activation (S100B and YKL-40) showed the strongest correlation with S and P CSF levels. Additionally, both CSF S and P levels were significantly increased in AD patients compared to MCI patients and HC, respectively. Higher plasma P levels were observed in AD patients compared to HC [62] and associated with an increased risk of dementia [76]. However, Park et al. showed that serum P levels negatively correlate with cerebral Aβ deposition [77]. To our knowledge, no other study analyzed S levels in AD patients, although the intake of sublimed sulfur was suggested to be protective in AD [78]. Most studies analyzed sulfur-containing compounds in AD patients (reviewed in [79]). Thus, plasma sulfate levels were significantly decreased in 10 AD patients compared to HC [80]. A recent study suggested that the intake of hydrogen sulfide (H_2_S) is beneficial in AD [81], whereas Disbrow et al. observed an increase in H_2_S levels in AD patients [82]. Additionally, H_2_S can be produced by some bacteria [83] that have been associated with a higher risk of AD (such as *Porphyromonas gingivalis* and *Helicobacter pylori*; reviewed in [84]). Damage of the BBB during AD pathogenesis can facilitate pathogen entry into the brain; through this route, such pathogens may contribute to neuroinflammation, a key feature of AD [85]. Whether the association between CSF S levels and of S100B and YKL-40 represent an indicator of microbial infections that contribute to AD pathogenesis needs further investigation.

The strength of our study is in the analysis of 24 macro- and microelements measured in CSF and 21 measured in plasma, in addition to 11 CSF AD biomarkers determined in nearly 200 participants. Only two studies [29,32] that investigated the association of macro- and microelements with CSF AD biomarkers included more participants than our current study. We used different statistical methods to test the association of macro- and microelements with CSF AD biomarkers, including redescription mining. Only one recent study used machine learning to classify AD, MCI patients, and HC using CSF Fe and CSF Aβ_1–42_, p-tau, and t-tau (overall 69 participants) [86]. A limitation of our study is the lack of information on possible confounding variables, such as smoking habits (especially in regard to Cd levels) and intake of over-the-counter dietary supplements (especially regarding essential metals and non-metals).

In conclusion, our study showed that essential metals (Ca, Co, Cu, Fe, Mg, Mn, Mo, Na, K, and Zn), heavy metals (As, Cd, Hg, Ni, Pb, and Tl), and essential non-metals (P, S, and Se) are positively associated with CSF AD biomarkers, mainly phosphorylated tau isoforms, VILIP-1, S100B, NFL, and YKL-40, suggesting new diagnostic opportunities and therapeutic targets in future studies on AD.

## 4. Materials and Methods

### 4.1. Participants and Sample Collection

We included 193 patients who were admitted to the University Hospital Center Zagreb and General Hospital Varaždin. Patients underwent thorough neurological testing, including MMSE, complete blood tests (albumin levels, thyroid function, levels of vitamin B12 and electrolytes), and VDRL testing for syphilis, as described previously [87]. NINCDS-ADRDA criteria for AD were fulfilled by 124 patients [88], while 50 patients fulfilled the criteria for MCI [88,89], and 19 were HC. CSF samples were collected from all participants by lumbar puncture (performed at intervertebral spaces L3/L4 or L4/L5). After centrifugation at 2000× *g* for 10 min, CSF samples were aliquoted in polypropylene tubes and stored at −80 °C. Venous blood samples were collected from 143 participants in the morning on an empty stomach. Samples were collected using plastic syringes (with 1 mL of acid citrate dextrose as an anticoagulant). Thrombocyte-free plasma was collected by centrifugation, first at 1100× *g* for 3 min and then at 5087 g for 15 min. Plasma samples were stored at −20 °C. Supplementary Table S1 summarizes demographic data. All procedures were approved by the Ethical Committee of the Clinical Hospital Center Zagreb (protocol no. 02/21 AG, class 8.1–18/82-2 from 24 April 2018) and the Central Ethical Committee of the University of Zagreb Medical School (protocol no. 380-59-10106-18-111/126, class 641-01/18-02/01 from 20 June 2018).

### 4.2. Analysis of Macro- and Microelements by Inductively Coupled Plasma Mass Spectroscopy

Inductively coupled plasma mass spectroscopy (ICP-MS) was used for the measurement of CSF and plasma levels of As, B, Ca, Cd, Co, Cu, Fe, Hg, Li, Mg, Mn, Mo, Na, Ni, P, Pb, S, Se, Sr, Tl, and Zn, and CSF levels of Al, Ba, and K (Supplementary Table S2). Cr levels were also measured in CSF and plasma, but due to possible contamination of the samples with Cr from the needles used for sample collection, it was excluded from statistical analysis. ICP-MS was performed on Agilent 7500cx (Agilent Technologies, Tokyo, Japan). Before the analysis, CSF samples were diluted at 1:10, while plasma samples were diluted at 1:20 with a solution containing 0.01 mM EDTA, 0.07% (*v*/*v*) Triton X-100, 0.7 mM ammonia, and 2 µg/L of internal standards (Ge, Rh, Tb, Lu, and Ir) in ultrapure water. We used a MicroMist nebulizer combined with a Peltier standard quartz spray chamber (Scott–type, cooled at 2 °C) and a quartz torch with a 2.5-mm diameter injector with a Shield Plate system and Ni sampler and skimmer cones. Daily optimization of ICP-MS working conditions was achieved using a tuning solution of 1 µg/L ^7^Li, ^59^Co, ^89^Y, ^140^Ce, and ^205^Tl. HVAC systems (Heating, Ventilating, and Air Conditioning) combined with HEPA filters were used for sample preparation and analysis. Quantification of elements concentration in samples was done by the standard addition method (matrix-matched calibration). Commercially available reference materials were used to confirm the accuracy of the measurements: ClinChek Serum Controls (Level I and II) and ClinChek Plasma Controls (Level I and II) from RECIPE (Munich, Germany); Seronorm Trace Elements Serum (Level I and II) (Sero AS, Billingstad, Norway).

### 4.3. Analysis of AD Biomarkers in CSF

Eleven AD biomarkers were measured in CSF by the enzyme-linked immunosorbent assays (ELISA); Aβ_1–42_, VILIP-1, t-tau, p-tau_181_, p-tau_231_, tau phosphorylated at Ser 199 (p-tau_199_), NFL, S100B, YKL-40, PAPP-A, and albumin. Following ELISA kits were used for the measurement of CSF AD biomarkers; Aβ_1–42_ (Innotest β-amyloid1–42, Fujirebio, Gent, Belgium), p-tau_181_ (Innotest Phospho-Tau (181P), Fujirebio), p-tau_231_ (Tau (pT231) Phospho-ELISA Kit, Human, Thermo Fisher Scientific, Waltham, MA, USA), p-tau_199_ (TAU [pS199] Phospho-ELISA Kit, Human, Thermo Fisher Scientific), t-tau (Innotest hTau Ag, Fujirebio), VILIP-1 (VILIP-1 Human ELISA, BioVendor, Brno, Czech Republic), NFL (Human NF-L/NEFL ELISA Kit, LifeSpan BioSciences, Seattle, WA, USA), S100B (Human S100B, R&D Systems, Minneapolis, MN, USA), YKL-40 (Chitinase 3-like 1 Quantikine ELISA Kit R&D Systems), PAPP-A (Human Pappalysin-1/PAPP-A Quantikine ELISA Kit, R&D Systems), and albumin (Human Albumin ELISA Kit, Abcam, Cambridge, UK).

### 4.4. Statistical Analysis

#### 4.4.1. Correlation and Principal Component Analysis

Correlation (Spearman’s and Pearson’s correlation coefficient) and linear regression were used for testing the association between CSF AD biomarkers and macro- and microelements measured in CSF and plasma. Additionally, principal component analysis (PCA, unsupervised machine learning method) was used to reduce the dimensionality of macro- and microelements measured in CSF and plasma. PCA analyses were performed for the macro- and microelements measured in CSF, and for the macro- and microelements measured in plasma. Orthogonal (varimax) rotation was used in PCA, while the adequacy of test items and sample size for factor analysis was assessed by Bartlett’s χ^2^ test of sphericity and the Kaiser–Meyer–Olkin index. The list of 23 macro- and microelements measured in CSF (we excluded P from the PCA because it was measured in a relatively small number of participants) and 21 macro- and microelements measured in plasma were reduced to key factors (groups). If a factor loading for an element was ≥0.4, it was considered that the element loads in the given group (factor). Factor loading measures the correlation between the element and a factor. Additionally, factor-specific scores were calculated for each individual. Multiple linear regression analyses were used for testing the association between PCA-obtained macro- and microelements’ groups (factors) and CSF AD biomarkers (with CSF AD biomarkers as dependent variables and macro- and microelements’ groups as independent variables). Statistical analyses were performed using SPSS 19.0.1 (SPSS Inc., Chicago, IL, USA) and R (R Foundation for Statistical Computing, Vienna, Austria), with the α value set at 0.05 for statistical significance.

#### 4.4.2. Redescription Mining

Three redescription sets were created using the redescription mining algorithm CLUS-RM [90,91]. The first redescription set describes patients that share properties of various CSF AD biomarkers and macro- and microelements measured in CSF. The second redescription set describes patients that share properties of various CSF AD biomarkers and macro- and microelements measured in plasma. The third redescription set describes patients that share properties of various CSF AD biomarkers with macro- and microelements measured in CSF and macro- and microelements measured in plasma. All three discovered redescription sets enable association analyses between indicators from the three groups of attributes: (a) CSF AD biomarkers, (b) macro- and microelements measured in CSF, and (c) macro- and microelements measured in plasma. 

In all experiments, we performed 10 runs with different random initialization and 30 iterations of the CLUS-RM algorithm for each run. In each run, the algorithm creates one starting initial clustering of patients that is used to create initial pair of Predictive Clustering trees (PCTs). Initial pairs of PCTs are used as a starting point for a sequence of iterations (called alternations) that create two pairs of matching PCTs per iteration, used to construct redescriptions. A supplement random forest, consisting of twenty trees and a conjunctive refinement procedure was used to obtain more diverse and accurate redescriptions (for more details see [90,91]). The final result of the methodology is a set of redescriptions that are tuples of logical rules. Each redescription describes a set of patients (the support set of a redescription). A redescription describes a patient if every rule from the corresponding tuple describes this patient, and a rule describes a patient if this patient has measurements and concentrations that satisfy logical conditions specified in the rule. For example, a rule PAPP-A (122.39–511.26) describes all patients whose measured PAPP-A level is in the interval [122.39, 511.26]. A rule PAPP-A (122.39–511.26) AND MMSE (5–28) AND AGE (49–82) describes all patients that additionally have MMSE measured concentration in the interval (5, 28), are at least 49 years old and maximally 82 years old. 

In this work, we use a minimal support set size of 30 and a maximal support set size of 155 for the first set, a minimal ort set size of 30 and a maximal support set size of 115 for the second set, and a minimal support set size of 20 and a maximal support set size of 110 for the third set. Redescription accuracy measures what fraction of patients, described by either of the rules forming some redescription, is described by all these rules. The corresponding measure that captures this property is called the Jaccard index [92]. In this work, we use the minimal accuracy threshold of 0.5. The statistical significance of a redescription (reported through a corresponding *p*-value) measures how probable it would be to obtain a redescription at random (by a random choice of rules that form it), so that each rule in a randomly created redescription describes the same number of patients as the original, and that the resulting redescription has a support set size equal or larger to the support set size of the original redescription [93]. In this work, we use a maximal *p*-value of 0.01. To maximize interpretability and to allow analyses of strong associations between different indicators (measurements), we construct redescriptions with rules containing only logical AND operator. As it can be seen from the example above, when only AND operator is used, each patient described by a rule must have all measurements for all indicators in the exactly specified interval. On the other hand, the rule NOT PAPP-A (122.39–511.26) describes all patients such that either PAPP-A < 122.39 OR PAPP-A >511.26, and understanding such rules would be much more difficult.

## Figures and Tables

**Table 1:** Correlations of CSF AD biomarkers with macro- and microelements.

In CSF	p-tau_181_	p-tau_199_	p-tau_231_	VILIP-1	YKL-40	S100B	NFL	PAPP-A	Aβ_1–42_, Aβ_1–42_/p-tau_181_ or Albumin
**Fe**	r_S_ = 0.26, *p* < 0.001(193) *^B^	rs = 0.258, *p* < 0.001 (193) *^F^	rs = 0.236, *p* = 0.001 (193) *^C^	rs = 0.407, *p* < 0.001 (193)*	rs = 0.32, *p* < 0.001 (178)*	rs = 0.286, *p* < 0.001 (158) *^E^	rs = 0.344, *p* < 0.001 (118)*	rs = 0.243, *p* = 0.001 (178)*	rs = 0.314, *p* < 0.001 (142) (albumin)*
**Cu**	rs = 0.321, *p* < 0.001 (193)*	rs = 0.334, *p* < 0.001 (193)*	rs = 0.303, *p* < 0.001 (193)*	rs = 0.537, *p* < 0.001 (193)*	rs = 0.309, *p* < 0.001 (178)*	rs = 0.483, *p* < 0.001 (158)*	rs = 0.323, *p* < 0.001 (118)*	rs = 0.435, *p* < 0.001 (178)*	rs = 0.439, *p* < 0.001 (142) (albumin)*
**S**		rs = 0.246, *p* = 0.001 (193) *^A^	rs = 0.322, *p* < 0.001 (193) *^A^	rs = 0.549, *p* < 0.001 (193)*	rs = 0.394, *p* < 0.001 (178)*	rs = 0.611, *p* < 0.001 (159)*	rs = 0.381, *p* < 0.001 (119)*	rs = 0.523, *p* < 0.001 (178)*	rs = 0.49, *p* < 0.001 (142) (albumin)* r = −0.25, *p* < 0.001 (192) (Aβ_1–42_/p-tau_181_) *^B^
**K**	r_S_ = 0.239, *p* = 0.001 (193) *^G^	rs = 0.27, *p* < 0.001 (193)*		rs = 0.541, *p* < 0.001 (193)*	rs = 0.26, *p* < 0.001 (178) *^C^	rs = 0.311, *p* < 0.001 (159)*	rs = 0.302, *p* = 0.001 (119)*	rs = 0.288, *p* < 0.001 (178)*	
**Se**	rs = 0.466, *p* < 0.001 (193)*	rs = 0.399, *p* < 0.001 (193)*	rs = 0.344, *p* < 0.001 (193)*	rs = 0.614, *p* < 0.001 (193)*	rs = 0.277, *p* < 0.001 (178) *^H^	rs = 0.398, *p* < 0.001 (159)*		rs = 0.322, *p* < 0.001 (178)*	r = 0.294, *p* < 0.001 (142) (albumin) *^B^
**Co**	r_S_ = 0.223, *p* = 0.002 (193) *^B^		rs = 0.359, *p* < 0.001 (192)*	rs = 0.446, *p* < 0.001 (192)*	rs = 0.284, *p* < 0.001 (179) *^A^	rs = 0.42, *p* < 0.001 (159)*	rs = 0.315, *p* < 0.001 (119)*	rs = 0.476, *p* < 0.001 (177)*	r = 0.349, *p* < 0.001 (142) (albumin) *^A^
**Mn**	rs = 0.23, *p* = 0.001 (193) *^B^		rs = 0.261, *p* < 0.001 (193)*	rs = 0.33, *p* < 0.001 (193)*		rs = 0.274, *p* < 0.001 (159) *^B^		rs = 0.318, *p* < 0.001 (178)*	
**Ni (CSF)**	rs = 0.253, *p* < 0.001 (188)*		r = 0.307, *p* < 0.001 (188)*	rs = 0.309, *p* < 0.001 (188)*	rs = 0.252, *p* = 0.001 (178) *^C^			rs = 0.306, *p* < 0.001 (173)*	rs = 0.29, *p* < 0.001 (139) (albumin) *^C^
**Ni (plasma)**						r = 0.353, *p* < 0.001 (123)*		r = 0.376 *p* < 0.001 (135)*	rs = 0.288, *p* = 0.001 (134) (albumin)*
**Na (CSF)**		rs = 0.3, *p* < 0.001 (193) *^A^		rs = 0.590, *p* < 0.001 (193) *^A^	rs = 0.356, *p* < 0.001 (178)*	rs = 0.422, *p* < 0.001 (159)*	rs = 0.325, *p* < 0.001 (119)*	rs = 0.338, *p* < 0.001 (178)*	
**Na (plasma)**						r = 0.321, *p* < 0.001 (123)			
**Mg**		rs = 0.255, *p* < 0.001 (193) *^A^		rs = 0.550, *p* < 0.001 (193) *^A^	rs = 0.29, *p* < 0.001 (178)*	rs = 0.37, *p* < 0.001 (159)*	rs = 0.366, *p* < 0.001 (119)*	rs = 0.345, *p* < 0.001 (178)*	
**As**		rs = 0.384, *p* < 0.001 (192)*		rs = 0.521, *p* < 0.001 (192)*	rs = 0.32, *p* < 0.001 (177)*	rs = 0.344, *p* < 0.001 (157)*			
**Hg**	rs = 0.228, *p* = 0.001 (192)*		rs = 0.262, *p* < 0.001 (192)*	rs = 0.347, *p* < 0.001 (192)*			rs = 0.331, *p* < 0.001 (117)*		
**Tl**			r = 0.233, *p* = 0.001 (193)*	rs = 0.253, *p* < 0.001 (193)*		rs = 0.299, *p* < 0.001 (159)*	r = 0.334, *p* < 0.001 (119)*	rs = 0.37, *p* < 0.001 (178)*	
**P**					rs = 0.444, *p* < 0.001 (76)*	rs = 0.506, *p* < 0.001 (72)*	rs = 0.825, *p* < 0.001 (37)*	rs = 0.382, *p* < 0.001 (82)*	r_S_ = 0.414, *p* < 0.001 (89) (Aβ)*
**Li (CSF)**				rs = 0.227, *p* = 0.001 (193) *^A^	rs = 0.281, *p* < 0.001 (178)*	rs = 0.368, *p* < 0.001 (159) *^B^	rs = 0.290, *p* = 0.001 (119)*	rs = 0.318, *p* < 0.001 (178) *^A^	
**Li (plasma)**						r = −0.413, *p* < 0.001 (123)		r = −0.47 *p* < 0.001 (135)	rs = −0.287, *p* = 0.001 (134) (albumin) ^A^
**Zn**				r = 0.327, *p* < 0.001 (193)*		r = 0.376, *p* < 0.001 (158)*	r = 0.295, *p* = 0.001 (118)*	r = 0.322, *p* < 0.001 (178)*	r = 0.375, *p* < 0.001 (142) (albumin)
**Mo**				r = 0.408, *p* < 0.001 (193)*	r = 0.269, *p* < 0.001 (178)*	r = 0.29, *p* < 0.001 (159)*		r = 0.336, *p* < 0.001 (178)*	
**Ba**				r = 0.25, *p* < 0.001 (191) *^B^		r = 0.374, *p* < 0.001 (157)	r = 0.294, *p* = 0.001 (117) ^B^		
**Ca**				rs = 0.404, *p* < 0.001 (193)		rs = 0.295, *p* < 0.001 (159)*	rs = 0.342, *p* < 0.001 (119) *	r = 0.25, *p* = 0.001 (178)	
**B**				rs = 0.258, *p* < 0.001 (193) *^B^			rs = 0.299, *p* = 0.001 (119) *^B^		
**Pb**					r = −0.285, *p* < 0.001 (178)*				
**Sr**							r = 0.405, *p* < 0.001 (119)*		

Data are presented as either Spearman’s or Pearson’s correlation coefficients and *p*-values, with the number of participants given in the brackets; (* *p* ≤ 0.001). ^A^ Significance was lost after correction for the effect of age, gender, diagnosis, and duration of the disease. Statistically significant after correction for the confounding effect of: ^B^ age, gender, and diagnosis; ^C^ gender, diagnosis, and duration of the disease; ^D^ age and gender; ^E^ diagnosis and duration of the disease; ^F^ diagnosis; ^G^ gender; ^H^ gender and diagnosis. Aβ_1–42_, amyloid β_1–42_; CSF, cerebrospinal fluid; NFL, neurofilament light chain; PAPP-A, pregnancy-associated plasma protein A; p-tau_181_, tau protein phosphorylated at threonine 181; p-tau_231_, tau protein phosphorylated at threonine 231; p-tau_199_, tau protein phosphorylated at serine 199; S100B, S100 calcium-binding protein B; VILIP-1, Visinin-like protein 1; YKL-40, chitinase-3-like protein 1.

**Table 2:** CSF macro- and microelements and their factor loadings for 6 groups (factors) (given by the PCA).

Factors	CSF Macro- and Microelements (Factor Loadings)
Factor 1	As (0.470), Ba (0.611), Ca (0.845), Co (0.646), Cu (0.800), Fe (0.712), K (0.897), Mg (0.919), Mn (0.702), Na (0.870), Ni (0.562), S (0.748), Se (0.825), Sr (0.590), Tl (0.584), Zn (0.641)
Factor 2	Al (0.565), Cd (0.664), Pb (0.871)
Factor 3	B (0.595), Li (0.584)
Factor 4	None loaded
Factor 5	Hg (−0.505), Mo (0.601)
Factor 6	None loaded

**Table 3:** Plasma macro- and microelements and their factor loadings for 6 groups (factors) (given by the PCA).

Factors	Plasma Macro- and Microelements (Factor Loadings)
Factor 1	Ca (0.848), Co (0.491), Cu (0.59), Mg (0.659), Mn (0.577), Na (0.831), P (0.735), S (0.844), Se (0.728), Tl (0.35), Zn (0.569)
Factor 2	B (0.571), Cd (0.604), Li (0.649), Mo (0.580), Pb (0.38)
Factor 3	As (0.634), Hg (0.651)
Factor 4	Ni (0.617)
Factor 5	Sr (−0.595)
Factor 6	Fe (0.478)

**Table 4:** Results of linear regression analysis; comparison of factors obtained by PCA of CSF macro- and microelements with CSF AD biomarkers.

Factor	CSF AD Biomarker	Linear Regression Analysis
Factor 2 (Al, Cd, and Pb)	p-tau_181_	β = 0.321, SE = 3.064, *p* < 0.001
	p-tau_231_	β = 0.375, SE = 0.263, *p* < 0.001
	VILIP-1	β = 0.173, SE = 5.531, *p* = 0.007
	PAPP-A	β = 0.21, SE = 8.659, *p* = 0.005
	albumin	β = 0.231, SE = 12.494, *p* = 0.007
	Aβ_1–42_/p-tau_181_	β = −0.192, SE = 0.873, *p* = 0.009
Factor 1 (As, Ba, Ca, Co, Cu, Fe, K, Mg, Mn, Na, Ni, S, Se, Sr, Tl, and Zn)	p-tau_199_	β = 0.331, SE = 0.262, *p* < 0.001
	VILIP-1	β = 0.495, SE = 5.531, *p* < 0.001
	YKL-40	β = 0.205, SE = 7663.241, *p* = 0.007
	S100B	β = 0.24, SE = 30.022, *p* = 0.002
	PAPP-A	β = 0.148, SE = 7.893, *p* = 0.041
Factor 3 (B and Li)	S100B	β = 0.259, SE = 31.29, *p* = 0.001
	NFL	β = 0.285, SE = 6.257, *p* = 0.001
	PAPP-A	β = 0.275, SE = 8.551, *p* < 0.001
Factor 5 (Hg and Mo)	NFL	β = 0.312, SE = 6.388, *p* < 0.001

Aβ_1–42_, amyloid β; AD, Alzheimer’s disease; CSF, cerebrospinal fluid; NFL, neurofilament light chain; PAPP-A, pregnancy-associated plasma protein A; p-tau_181_, tau protein phosphorylated at threonine 181; p-tau_231_, tau protein phosphorylated at threonine 231; p-tau_199_, tau protein phosphorylated at serine 199; S100B, S100 calcium-binding protein B; VILIP-1, Visinin-like protein 1; YKL-40, chitinase-3-like protein 1.

**Table 5:** Results of linear regression analysis; comparison of factors obtained by PCA of plasma macro- and microelements with CSF AD biomarkers.

Factor	CSF AD Biomarker	Linear Regression Analysis
Factor 1 (Ca, Co, Cu, Mg, Mn, Na, P, S, Se, Tl, and Zn)	VILIP-1	β = −0.191, SE = 7.341, *p* = 0.028
Factor 2 (B, Cd, Li, Mo and Pb)	Aβ_1–42_/p-tau_181_	β = 0.192, SE = 0.873, *p* = 0.009
Factor 3 (As and Hg)	VILIP-1	β = 0.17, SE = 7.341, *p* = 0.05
	NFL	β = 0.237, SE = 6.109, *p* = 0.013
Factor 4 (Ni)	p-tau_231_	β = 0.224, SE = 0.39, *p* = 0.014
	VILIP-1	β = 0.222, SE = 7.341, *p* = 0.011
	S100B	β = 0.405, SE = 36.065, *p* < 0.001
	NFL	β = 0.295, SE = 6.450, *p* = 0.002
	PAPP-A	β = 0.428, SE = 8.945, *p* < 0.001
	albumin	β = 0.306, SE = 15.086, *p* = 0.001
Factor 5 (Sr)	p-tau_199_	β = 0.204, SE = 0.284, *p* = 0.023

Aβ_1–42_, amyloid β; AD, Alzheimer’s disease; CSF, cerebrospinal fluid; NFL, neurofilament light chain; PAPP-A, pregnancy-associated plasma protein A; p-tau_181_, tau protein phosphorylated at threonine 181; p-tau_231_, tau protein phosphorylated at threonine 231; p-tau_199_, tau protein phosphorylated at serine 199; S100B, S100 calcium-binding protein B; VILIP-1, Visinin-like protein 1; YKL-40, chitinase-3-like protein 1.

**Table 6:** Most significant redescriptions given by the combination of CSF AD biomarkers (first view—W1) and macro- and microelements measured in CSF (second view—W2).

Redescription Specific for:	HC (%)	MCI (%)	AD (%)	W1R	W2R	JS	*p*-Value
Macro- and Microelements in CSF							
**AD**	**5.3%**	**6%**	**36%**	VILIP-1 (121.28–366.14) MMSE (13–25) Aβ_1–42_ (275–1246)	As in CSF (0.34–2.62) S in CSF (14.3–40.2)	0.56977	1.548 × 10^−7^ *
**AD**	**5.3%**	**6%**	**31.2%**	VILIP-1 (121.28–366.14) MMSE (13–25) Aβ_1–42_ (275–1246)	As in CSF (0.34–2.62) K in CSF (120–243) S in CSF (14.3–40.2) Cd in CSF (0.01–0.033)	0.5584	5.711 × 10^−8^ *
**AD**	**21%**	**44%**	**70.4%**	YKL-40 (102,044.391955–893,335.0) VILIP-1 (62.52–366.14)	K in CSF (94.0–243)	0.7125	0.003 *
**AD**	**15.7%**	**38%**	**63.2%**	S100B (234.16–2325.45) VILIP-1 (62.52–366.14) MMSE (10–29)	Zn in CSF (33.0–318.1) K in CSF (94–243)	0.65584	0.002 *
**AD**	**15.8%**	**38%**	**63.2%**	S100B (234.16–2325.45) VILIP-1 (62.52–366.14) MMSE (10–29)	Mg in CSF (22.45–52.21) Zn in CSF (33–318.1)	0.66013	0.002 *
**AD&MCI**	**5.3%**	**46%**	**59.2%**	VILIP-1 (62.52–366.14) MMSE (10–29)	Na in CSF (2545–5335) Li in CSF (0.21–49.93) Cu in CSF (9.9–36.98)	0.64901	0.004 *
**AD&MCI**	**5.3%**	**38%**	**50.4%**	VILIP-1 (62.52–366.14) MMSE (10–29)	Se in CSF (1.1–3.35) Cu in CSF (10.7–36.27) Co in CSF (0.046–0.219) Ba in CSF (0.98–110.4) Cd in CSF (0.004–0.033)	0.60145	3.545 × 10^−4^ *
**AD&MCI**	**5.3%**	**34%**	**52%**	S100B (234.16–2325.45) VILIP-1 (62.52–366.14) MMSE (10–29)	Co in CSF (0.046–0.219) Se in CSF (1.1–3.35) Zn in CSF (27.9–246.1)	0.58865	0.003 *
**AD&MCI**	**5.3%**	**34%**	**51.2%**	S100B (234.16–2325.45) VILIP-1 (62.52–366.14) MMSE (10–29)	Li in CSF (0.21–49.93) Zn in CSF (33–318.1) Cu in CSF (10.2–36.98) Na in CSF (2545.0–5335)	0.59854	0.001 *
**AD&MCI**	**5.3%**	**42%**	**46.4%**	VILIP-1 (62.52–366.14) MMSE (10–29)	Se in CSF (1.1–3.35) Zn in CSF (27.9–111) Ba in CSF (0.62–105.83)	0.55944	0.003 *
**AD&MCI**	**5.3%**	**44%**	**60.8%**	VILIP-1 (62.52–366.14) MMSE (10–29)	Co in CSF (0.046–0.219) Se in CSF (1.1–3.35) Zn in CSF (27.9–246.1)	0.66	0.002 *
**AD&MCI**	**5.3%**	**38%**	**56%**	VILIP-1 (62.52–366.14) MMSE (10–29)	Cu in CSF (10.3–36.27) Co in CSF (0.046–0.219) Fe in CSF (19.1–111.3) S in CSF (12.7–32) Ba in CSF (1.61–110.4)	0.61644	0.002 *
**AD&MCI**	**5.3%**	**38%**	**56%**	VILIP-1 (62.52–366.14) MMSE (10–29)	Cu in CSF (10.3–36.27) Co in CSF (0.046–0.219) Fe in CSF (19.1–111.3) S in CSF (12.7–32) Ba in CSF (1.61–110.4)	0.616434	0.002 *
**AD&MCI**	**5.3%**	**36%**	**45.6%**	VILIP-1 (62.52–366.14) MMSE (10–29)	Se in CSF (1.1–3.69) Li in CSF (0.39–49.93) Ba in CSF (3.2–110.4)	0.55882	4.295 × 10^−4^ *
**AD&MCI**	**5.3%**	**38%**	**52.8%**	VILIP-1 (62.52–366.14) MMSE (10–29)	Se in CSF (1.1–3.35) Zn in CSF (27.9–246.1) Co in CSF (0.046–0.219) Cd in CSF (0.004–0.033)	0.6014	0.001 *
**AD&MCI**	**5.3%**	**34%**	**50.4%**	VILIP-1 (62.52–366.14) MMSE (10–29)	S in CSF (9.6–32) Se in CSF (1.1–3.35) Co in CSF (0.046–0.219) Ba in CSF (0.98–110.4) Cd in CSF (0.004–0.033)	0.59124	3.177 × 10^−4^ *
**AD&MCI**	**5.3%**	**34%**	**56.8%**	S100B (234.16–2325.45) VILIP-1 (62.52–366.14) MMSE (10–29)	Zn in CSF (28–246.1) Cu in CSF (9.92–36.27) Na in CSF (2063–4873) Co in CSF (0.046–0.219)	0.58940	0.01 *
**AD&MCI**	**5.3%**	**38%**	**56.8%**	VILIP-1 (62.52–366.14) MMSE (10–29)	Cu in CSF (10.3–36.27) Co in CSF (0.046–0.219) S in CSF (12.7–32) Ba in CSF (1.61–110.4)	0.61486	0.004 *
**AD&MCI**	**0%**	**36%**	**48.8%**	VILIP-1 (62.52–366.14) MMSE (10–29)	Hg in CSF (0.025–1.063) Cu in CSF (10.3–36.16) Co in CSF (0.046–0.219) Fe in CSF (19.1–111.3) S in CSF (12.7–32) Ba in CSF (1.61–110.4)	0.5411	0.009 *
**AD&MCI**	**5.3%**	**30%**	**44%**	S100B (234.16–2325.45) VILIP-1 (62.52–366.14) MMSE (10–29)	Se in CSF (1.1–3.35) Cu in CSF (10.7–36.27) Co in CSF (0.046–0.219) Ba in CSF (0.98–110.4) Cd in CSF (0.004–0.033)	0.568	2.255 × 10^−4^ *
**AD&MCI**	**5.3%**	**36%**	**48%**	S100B (234.16–2325.45) VILIP-1 (62.52–366.14) MMSE (10–29)	Li in CSF (0.25–49.93) Zn in CSF (33–318.1) Cu in CSF (10.2–36.98) Na in CSF (2196–5335)	0.57246	0.002 *
**AD&MCI**	**5.3%**	**30%**	**44.8%**	S100B (234.16–2325.45) VILIP-1 (62.52–366.14) MMSE (10–29)	Se in CSF (1.1–3.35) Zn in CSF (27.9–246.1) Co in CSF (0.046–0.219) Cd in CSF (0.004–0.033)	0.54545	0.002 *
**AD&MCI**	**5.3%**	**26%**	**50.4%**	S100B (234.16–2325.45) VILIP-1 (62.52–366.14) MMSE (10–29)	Zn in CSF (33–246.1) Cu in CSF (10.3–36.27) Co in CSF (0.046–0.219) S in CSF (12.7–32) Ba in CSF (1.61–110.4)	0.56618	0.002 *
**AD&MCI**	**15.8%**	**52%**	**76%**	VILIP-1 (62.52–366.14) MMSE (10–29)	K in CSF (94–243)	0.77019	0.002 *
**AD&MCI**	**15.8%**	**54%**	**76.8%**	VILIP-1 (62.52–366.14) MMSE (10–29)	Na in CSF (2527–5492)	0.76829	0.003 *
**AD&MCI**	**15.8%**	**46%**	**68%**	VILIP-1 (62.52–366.14) MMSE (10–29)	Se in CSF (12.7–40.8)	0.70253	0.005 *
**AD&MCI**	**15.8%**	**52%**	**71.2%**	VILIP-1 (62.52–366.14) MMSE (10–29)	Se in CSF (1.1–3.69) Zn in CSF (27.9–318.1)	0.78146	1.676 × 10^−4^ *
**AD&MCI**	**15.8%**	**44%**	**66.4%**	VILIP-1 (62.52–366.14) MMSE (10–29)	Cd in CSF (0.004–0.033) Mg in CSF (22.45–52.21) Fe in CSF (15.5–116.3)	0.71053	0.001 *
**AD&MCI**	**15.8%**	**56%**	**78.4%**	VILIP-1 (62.52–366.14) MMSE (10–29)	Cu in CSF (9.35–36.98) S in CSF (7.8–40.8)	0.7771	0.004 *
**AD&MCI**	**15.8%**	**52%**	**75.2%**	VILIP-1 (62.52–366.14) MMSE (10–29)	Mg in CSF (22.45–52.21) S in CSF (7.9–40.8)	0.76398	0.002 *
**AD&MCI**	**15.8%**	**40%**	**60%**	S100B (234.16–2325.45) VILIP-1 (62.52–366.14) MMSE (10–29)	Zn in CSF (33–318.1) Cu in CSF (9.92–36.98) Fe in CSF (15.5–116.3) Al in CSF (0.9–6.75) Na in CSF (2063–5335)	0.65772	0.001 *
**AD&MCI**	**15.8%**	**42%**	**64.8%**	S100B (234.16–2325.45) VILIP-1 (62.52–366.14) MMSE (10–29)	Na in CSF (2063–5492) Zn in CSF (28–318.1) Cu in CSF (9.92–36.98)	0.67308	0.002 *
**AD&MCI**	**10.5%**	**34%**	**50.4%**	VILIP-1 (62.52–366.14) YKL-40 (102,044.391955–893,335.0) Aβ_1–42_ (272.94–1568.0)	Li in CSF (0.21–49.93) Se in CSF (0.89–3.69) Cu in CSF (10.7–36.98) K in CSF (94–243) Ba in CSF (1.61–110.4)	0.63077	1.050 × 10^−4^ *
**HC&MCI**	**47.4%**	**42%**	**28.8%**	*APOE* genotype = ɛ3ɛ3 Aβ_1–42_ (275–1672.36)	Pb in CSF (0.56–14.83) Ba in CSF (0.98–110.4)	0.51163	0.001 *
**HC&MCI**	**42.1%**	**50%**	**18.4%**	VILIP-1 (17.2–89.68)	K in CSF (37–134) S in CSF (4.2–17.6)	0.51852	4.439 × 10^−5^ *
**HC&MCI**	**42.1%**	**50%**	**15.2%**	VILIP-1 (17.2–89.68) p-tau_231_ (0–1.978) t-tau (44–598.0)	Se in CSF (0.36–1.97) K in CSF (37–134) S in CSF (4.2–17.6)	0.5	3.855 × 10^−5^ *
**HC&MCI**	**47.4%**	**30%**	**14.4%**	VILIP-1 (17.2–89.68)	Se in CSF (0.36–1.09) Mn in CSF (0.4–3.58)	0.53846	5.472 × 10^−8^ *
**HC&MCI**	**52.6%**	**56%**	**33.6%**	VILIP-1 (17.2–121.08) p-tau_181_ (16–145.9)	As in CSF (0.05–0.33) Fe in CSF (8.8–74.1)	0.625	7.274 × 10^−5^ *
**HC&MCI**	**47.4%**	**30%**	**12.8%**	VILIP-1 (17.2–89.68) p-tau_231_ (0–1.978) t-tau (44–598)	Mn in CSF (0.4–3.58) Se in CSF (0.36–1.09)	0.53333	6.249 × 10^−8^ *
**HC&MCI**	**42.1%**	**50%**	**16%**	VILIP-1 (17.2–89.68) p-tau_231_ (0–1.978) t-tau (44–598)	Se in CSF (0.36–1.97) K in CSF (37–140) S in CSF (4.2–17.7)	0.50476	3.487 × 10^−5^ *
**HC&MCI**	**47.4%**	**30%**	**13.6%**	t-tau (44–598) VILIP-1 (17.2–89.68)	Mn in CSF (0.4–3.58) Se in CSF (0.36–1.09)	0.53947	4.804 × 10^−8^ *
**HC&MCI**	**42.1%**	**50%**	**19.2%**	VILIP-1 (17.2–89.68)	K in CSF (37–140) S in CSF (4.2–17.7) Se in CSF (0.36–1.97)	0.53774	1.584 × 10^−5^ *
**HC&MCI**	**42.1%**	**50%**	**16%**	VILIP-1 (17.2–89.68) t-tau (44.0–598)	Se in CSF (0.36–1.97) K in CSF (37–134) S in CSF (4.2–17.6) Na in CSF (958–3658.0)	0.50962	3.108 × 10^−5^ *
**HC&MCI**	**42.1%**	**50%**	**17.6%**	VILIP-1 (17.2–89.68) t-tau (44–598)	Se in CSF (0.36–1.97) K in CSF (37–140) S in CSF (4.2–17.7)	0.52381	1.910 × 10^−5^ *
**HC&MCI**	**42%**	**50%**	**18.4%**	VILIP-1 (17.2–89.68)	Se in CSF (0.36–1.97) K in CSF (37–140) S in CSF (4.2–17.7)	0.53333	1.682 × 10^−5^ *
**HC**	**42.1%**	**22%**	**12.8%**	PAPP-A (23.64–345.15) VILIP-1 (17.2–89.12) p-tau_231_ (0–1.978) p-tau_199_ (0–5.781)	Se in CSF (0.36–1.09) Mn in CSF (0.4–3.58)	0.5303	3.716 × 10^−8^ *
**HC**	**42.1%**	**24%**	**12.8%**	VILIP-1 (17.2–89.12) p-tau_231_ (0–1.978) PAPP-A (23.64–345.15)	Mn in CSF (0.4–3.58) Se in CSF (0.36–1.09)	0.51429	1.133 × 10^−7^ *
**HC**	**42.1%**	**22%**	**12.8%**	VILIP-1 (17.2–89.12) p-tau_231_ (0–1.978) p-tau_199_ (0–5.781) PAPP-A (23.64–345.15)	Mn in CSF (0.4–2.51) Se in CSF (0.36–1.09) As in CSF (0.06–0.34)	0.55556	7.250 × 10^−9^ *

Data are presented as percentages of diagnoses and ranges of CSF biomarkers and elements. W1R refers to the first redescription query (constructed using attributes from the first view—W1), W2R refers to the second redescription query (constructed using attributes from the second view—W2), JS refers to the Jaccard similarity coefficient (Jaccard index) and *p*-value refers to the statistical significance of a redescription [22]. Aβ_1–42_, amyloid β; AD, Alzheimer’s disease; APOE, apolipoprotein E; CSF, cerebrospinal fluid; HC, healthy control; JS, Jaccard similarity index; MCI, mild cognitive impairment; MMSE, Mini-Mental State Examination; NFL, neurofilament light chain; PAPP-A, pregnancy-associated plasma protein A; p-tau_181_, tau protein phosphorylated at threonine 181; p-tau_231_, tau protein phosphorylated at threonine 231; p-tau_199_, tau protein phosphorylated at serine 199; S100B, S100 calcium-binding protein B; VILIP-1, Visinin-like protein 1; YKL-40, chitinase-3-like protein 1.* *p* ≤ 0.05.

**Table 7:** Most significant redescriptions given by the combination of CSF AD biomarkers (first view—W1) with macro- and microelements measured in plasma (second view—W2), and macro- and microelements measured both in CSF and in plasma (second view—W2 and third view W3).

Redescription Specific for:	HC (%)	MCI (%)	AD (%)	W1R	W2R		JS	*p*-Value
Macro- and Microelements in Plasma								
**AD**	**0%**	**8.6%**	**30.1%**	Albumin (58.44–411) PAPP-A (122.39–511.26) MMSE (5–28) Age (49–82) p-tau_181_ (23.19–126.71) T-tau (313.77–1992)	Ca in plasma (67.9–87.5) Na in plasma (3713–4033) Li in plasma (15–48) Cd in plasma (0.02–0.07) Ni in plasma (1.33–2.24)		0.64583	2.475 × 10^−8^ *
**AD**	**0%**	**8.6%**	**32.3%**	Albumin (58.44–411) S100B (293.15–1596.64) PAPP-A (141–529.35) MMSE (5–28) Age (53–82) p-tau_181_ (23.19–126.71) t-tau (207.85–1992)	Li in plasma (14–29) Sr in plasma (16.1–44.7) Cu in plasma (779–1456) Ca in plasma (67.4–85.3) Pb in plasma (102.8–150) Hg in plasma (0.04–1.51)		0.6346	2.9642 × 10^−8^ *
**AD**	**6.6%**	**22.9%**	**50.5%**	t-tau (207.85–1992) YKL-40 (4000–298,634)	Ca in plasma (73.6–90.4) Mg in plasma (22.3–43.6) Na in plasma (3131–4033) B in plasma (12.3–70.6) Mn in plasma (0.78–2.35)		0.57143	0.002 *
**AD**	**0%**	**5.7%**	**32.3%**	Albumin (58.44–413.96) S100B (293.15–1925.01) PAPP-A (118.65–529.35) MMSE (5–28) p-tau_181_ (23.19–126.71) T-tau (211–1992) Aβ_1–42_ (136.3–1230.43)	Na in plasma (3716–4023) Li in plasma (15–32) Ca in plasma (67.3–87.5)		0.51613	4.924 × 10^−6^ *
**AD**	**0%**	**14.3%**	**41.9%**	PAPP-A (122.39–529.35) YKL-40 (4000–364,460) MMSE (15–28) p-tau_231_ (0.432–25.83) T-tau (77.46–1992)	Li in plasma (14–32) Cd in plasma (0.01–0.07) Se in plasma (41.5–95.4) Mn in plasma (0.85–2.35)		0.53659	2.812 × 10^−4^ *
**AD**	**6.6%**	**8.6%**	**40.9%**	PAPP-A (122.39–529.35) YKL-40 (67,043.38–364,460) MMSE (5–28) Age (49–82) p-tau_181_ (23.19–127) T-tau (207.85–1992)	Ca in plasma (67.3–87.5) Mg in plasma (22.2–32.2) Na in plasma (3671–4039) Mo in plasma (0.72–12.02) As in plasma (0.52–24.8) Zn in plasma (522–942) Ni in plasma (1.33–4.09) Mn in plasma (0.83–2.35)		0.61111	6.127 × 10^−6^ *
**AD**	**0%**	**22.9%**	**41%**	T-tau (207.85–1992) MMSE (5–28) Age (49–82) p-tau_231_ (0.357–9.09) PAPP-A (122.39–529.35)	Cd in plasma (0–0.07) Ni in plasma (1.33–4.02) Mn in plasma (0.75–2.35) Ca in plasma (67.4–87.5) P in plasma (102.8–160.5) Na in plasma (3139–4033)		0.64474	5.877 × 10^−6^ *
**AD**	**6.6%**	**17.1%**	**41.9%**	PAPP-A (122.39–529.35) YKL-40 (4000–384,387.22) p-tau_231_ (0.432–25.83) p-tau_199_ (0–15.781) p-tau_181_ (21.6–319.16)	Li in plasma (14–32) Cd in plasma (0.01–0.07) Se in plasma (41.5–95.4) Mn in plasma (0.85–2.35)		0.51111	0.001 *
**AD**	**6.6%**	**22.9%**	**45.2%**	YKL-40 (4000–280,873) T-tau (313.77–2259) NFL (30.607–315.19)	Li in plasma (15–134) Mo in plasma (0.58–12.02) Mn in plasma (0.78–2.35) Ca in plasma (73.6–90.4) Mg in plasma (22.3–43.6) Na in plasma (3131–4033) B in plasma (12.3–70.6)		0.54839	0.001 *
**AD**	**6.6%**	**20%**	**45%**	PAPP-A (122.39–529.35) T-tau (313.77–1992)	Ca in plasma (67.4–87.5) Mg in plasma (22.3–32.2) Na in plasma (3215–4033) B in plasma (12.3–70.6) Hg in plasma (0.04–3.25) Mo in plasma (0.72–12.02) Zn in plasma (522–942) Ni in plasma (1.11–4.09) Mn in plasma (0.8–2.35)		0.54945	8.267 × 10^−4^ *
**AD&MCI**	**6.6%**	**45.7%**	**45.2%**	S100B (130.89–1267.47) Age (49–83) NFL (42.473–266.028)	Mo in plasma (0.83–12.02) Pb in plasma (103.9–223.7) As in plasma (0.48–3.2)		0.56731	0.006 *
**AD&MCI**	**0%**	**22.8%**	**41.9%**	t-tau (207.85–1992) MMSE (5–28) Age (49–82) p-tau_181_ (23.19–126.71) PAPP-A (122.39–529.35)	Cd in plasma (0.01–0.07) Ni in plasma (1.33–4.02) Mn in plasma (0.83–2.35) Ca in plasma (67.4–85.1) Pb in plasma (102.8–160.5)		0.63514	4.880 × 10^−6^ *
**AD&MCI**	**6.6%**	**48%**	**43%**	S100B (130.89–1267.47) Age (49–83) NFL (42.473–266.028)	Se in plasma (70.2–169.9) Co in plasma (0.26–1.07) Pb in plasma (103.9–223.7)		0.55769	0.007 *
**AD&MCI**	**6.6%**	**31.4%**	**53.8%**	T-tau (235–2259) YKL-40 (4000–298634) MMSE (5–28)	Pb in plasma (0.12–1.21) Mn in plasma (0.75–2.35) Fe in plasma (742–3939) B in plasma (12.3–56.6)		0.59048	0.008 *
**AD&MCI**	**6.6%**	**28.6%**	**47.3%**	PAPP-A (132.89–529.35) p-tau_181_ (21.6–319.16) p-tau_199_ (0.44–17.266) Aβ_1–42_ (136.3–696.0)	Mg in plasma (18.6–30.3) B in plasma (12.3–64.4) Pb in plasma (0.18–3.36) Cd in plasma (0.01–0.07) Zn in plasma (522–942) Ni in plasma (0.95–4.09) Mn in plasma (0.8–3.24) Ca in plasma (66.4–87.5)		0.56122	0.002 *
**AD&MCI**	**6.6%**	**40%**	**44.1%**	NFL (42.473–266.028) S100B (130.89–1267.47) PAPP-A (23.64–312.85) Age (49–83)	Li in plasma (16–174) P in plasma (103.9–249.4) As in plasma (0.48–24.8) Mg in plasma (21.8–43.6) Hg in plasma (0.04–2.23)		0.58333	0.001 *
**AD&MCI**	**6.6%**	**31.4%**	**46.2%**	PAPP-A (132.89–529.35) p-tau_181_ (21.6–319.16) Aβ_1–42_ (136.3–696)	Ca in plasma (66.4–85.3) Mg in plasma (20.8–30.3) B in plasma (12.3–70.6) Cd in plasma (0.01–0.07) Mo in plasma (0.72–12.02) Zn in plasma (522–942) Cu in plasma (500–1120) Ni in plasma (1.11–4.09) Co in plasma (0.26–1.07)		0.55556	0.003 *
**Macro- and Microelements in CSF and Plasma**								
**Redescription specific for:**	**HC (%)**	**MCI (%)**	**AD (%)**	W1R	W2R	W3R	JS	*p*-Value
**AD&MCI**	**6.6%**	**45.7%**	**66%**	Age (60–91) t-tau (148.21–1992)	Zn in plasma (507–942) S in plasma (654–896) Na in plasma (3248–4043) B in plasma (15.5–213.1)	Se in CSF (0.69–3.69)	0.59848	0.003 *
**AD&MCI**	**6.6%**	**45.7%**	**60.2%**	NFL (22–220.86) YKL-40 (81,665–483,135.59) p-tau_231_ (0.357–22.87) p-tau_181_ (23.19–319.16)	B in plasma (12.3–70.6) Co in plasma (0.27–1.07) P in plasma (78.1–249.4) Pb in plasma (0.1–3.36)	Se in CSF (0.74–3.69) Li in CSF (0.03–8.36)	0.54074	1.933 × 10^−4^ *
**AD&MCI**	**6.6%**	**40%**	**61.3%**	Albumin (73.84–1683.65) Age (64–91) p-tau_181_ (37.62–319.16)	S in plasma (452–945) Zn in plasma (492–1115)	Co in CSF (0.081–0.872)	0.5	0.009 *
**AD&MCI**	**6.6%**	**54.3%**	**72%**	Age (63–91) p-tau_181_ (23.19–319.16)	Pb in plasma (0.09–3.36) Mn in plasma (0.82–3.22) Li in plasma (16–174)	Se in CSF (0.82–3.69)	0.64444	8.697 × 10^−4^ *
**AD&MCI**	**6.6%**	**45.7%**	**64.6%**	Age (60–91) t-tau (148.21 <= 1992)	Ca in plasma (67.4–85.1) Mg in plasma (22.2–32.2) Na in plasma (3149–4039)	Se in CSF (0.69–3.69)	0.50327	0.009 *
**AD&MCI**	**6.6%**	**45.7%**	**72%**	Age (60–91) p-tau_231_ (0.278–25.83)	Na in plasma (2578–4012) B in plasma (15.5–213.1) Pb in plasma (0.1–3.36)	Li in CSF (0.05–21.32) Cu in CSF (10.6–36.98)	0.5283	0.002 *
**AD&MCI**	**26%**	**65.7%**	**79.6%**	p-tau_231_ (0.278–25.83) t-tau (88–2259)	Na in plasma (3149–4769) Mo in plasma (0.81–12.02)	Mg in CSF (13.92–42.7) Se in CSF (0.69–3.15)	0.66887	0.004 *
**AD&MCI**	**20%**	**65.7%**	**75.4%**	p-tau_231_ (0.278–25.83) t-tau (88–2259)	Na in plasma (3149–4023) Pb in plasma (0.09–3.36) Mo in plasma (0.81–12.02)	Cu in CSF (8.77–36.98)	0.63576	0.009 *
**AD&MCI**	**20%**	**65.7%**	**75.3%**	p-tau_231_ (0–7.941) p-tau_181_ (21.27–194) Aβ_1–42_ (136.3–1347)	B in plasma (7.9–70.6) Hg in plasma (0.04–3.25)	Ni in CSF (0.26–2.03) Fe in CSF (11.9–111.3)	0.70073	0.006 *
**AD&MCI**	**20%**	**60%**	**75.3%**	p-tau_231_ (0.288–25.83) t-tau (88–2259)	Ca in plasma (63.4–85.1) Mo in plasma (0.81–12.02) Zn in plasma (507–1115)	Mg in CSF (13.92–42.7) Se in CSF (0.69–3.15)	0.62667	0.009 *
**AD&MCI**	**20%**	**51.4%**	**74.2%**	Age (63–91) p-tau_181_ (23.19–319.16)	Mg in plasma (15.4–30.4) Mn in plasma (0.51–3.22)	Se in CSF (0.82–3.69)	0.65217	0.004 *
**HC**	**93.3%**	**71.4%**	**63.4%**	VILIP-1 (17.2–224.29)	Sr in plasma (14.6–44.7) Ca in plasma (71.4–108.2)	Cd in CSF (0.005–0.045) Na in CSF (958.0–4481.0)	0.64474	0.006 *

Data are presented as percentages of diagnoses and ranges of CSF biomarkers and elements. W1R refers to the first redescription query (constructed using attributes from the first view—W1), W2R refers to the second redescription query (constructed using attributes from the second view—W2), W3R refers to the third redescription query (constructed using attributes from the third view—W3), JS refers to the Jaccard similarity coefficient (Jaccard index) and *p*-value refers to the statistical significance of a redescription [22]. Aβ_1–42_, amyloid β; AD, Alzheimer’s disease; CSF, cerebrospinal fluid; HC, healthy control; JS, Jaccard similarity index; MCI, mild cognitive impairment; MMSE, Mini-Mental State Examination; NFL, neurofilament light chain; PAPP-A, pregnancy-associated plasma protein A; p-tau_181_, tau protein phosphorylated at threonine 181; p-tau_231_, tau protein phosphorylated at threonine 231; p-tau_199_, tau protein phosphorylated at serine 199; S100B, S100 calcium-binding protein B; VILIP-1, Visinin-like protein 1; YKL-40, chitinase-3-like protein 1.* *p* ≤ 0.05.

## Data Availability

All data are presented in this article. Original data are available from the corresponding author upon reasonable request.

## Supplementary Materials

The following supporting information can be downloaded at: https://www.mdpi.com/article/10.3390/ijms24010467/s1.

## Abbreviations

Aβ_1–42_, amyloid β_1–42_; Al, aluminum; ApoE, apolipoprotein E; APP, amyloid precursor protein; As, arsenic; B, boron; Ba, barium; BBB, blood–brain barrier; Ca, calcium; Co, cobalt; Cu, copper; CAA, cerebral amyloid angiopathy; Cd, cadmium; Cs, cesium; CSF, cerebrospinal fluid; DOHaD, developmental origins of health and disease; ELISA, enzyme-linked immunosorbent assay; Fe, iron; HC, healthy controls; Hg, mercury; ICP-MS, inductively coupled plasma mass spectroscopy; JS, Jaccard similarity coefficient; K, potassium; Li, lithium; MCI, mild cognitive impairment; Mg, magnesium; MMSE, Mini-Mental State Examination; Mn, manganese; Mo, molybdenum; Na, sodium; NFL, neurofilament light chain; NFT, neurofibrillary tangles; Ni, nickel; P, phosphorus; PAPP-A, pregnancy-associated plasma protein A, pappalysin-1; Pb, lead; PCA, principal component analysis; PTCs, Predictive Clustering trees; p-tau_181_, tau phosphorylated at Thr 181; p-tau_231_, tau phosphorylated at Thr 231; p-tau_199,_ tau phosphorylated at Ser 199; S, sulfur; SCI, subjective cognitive impairment; Se, selenium; SP, senile plaques; Sr, strontium; S100B, S100 calcium-binding protein B; Tl, thallium; t-tau, total tau; VaD, vascular dementia; VILIP-1, visinin-like protein 1; W1R, first redescription query; W2R, second redescription query; W3R, third redescription query; YKL-40, chitinase-3-like protein 1; Zn, zinc.
