# Supplementary material for: Heavy Metals and Essential Metals Are Associated with Cerebrospinal Fluid Biomarkers of Alzheimer’s Disease
Source: Int J Mol Sci. 2022 Dec 27;24(1):467. doi: 10.3390/ijms24010467 (PMC9820819; doi:10.3390/ijms24010467)
Supplement: Supplementary file 1 [file ijms-24-00467-s001.zip › ijms-2085606-supplementary.pdf]

Supplementary Table S1. Demographic data of included AD, MCI patients, and HC.

		AD	MCI	HC
N		124	50	19
Age	Median (25–75th percentile)	72 (65-78)	65 (59.8-74)	61 (51.8-75.3)
Sex	F/M	67/57	26/24	9/10
MMSE	Mean ± SD	20.5 ± 4.5	25.6 ± 2.8	28.4 ± 2.4
Disease duration (years)	Mean ± SD	2.5 ± 2.3	2.3 ± 2.6	-

AD, Alzheimer's disease; F, female; HC, healthy controls; M, male; MCI, mild cognitive impairment; MMSE, Mini-Mental State Examination.

Supplementary Table S2. CSF and plasma macro- and microelement levels in AD, MCI, and HC subjects.

Element		AD		MCI		HC		Differences between the groups
		Mean ± SD (Number of subjects)	Median (25-75th percentile)	Mean ± SD (Number of subjects)	Median (25-75th percentile)	Mean ± SD (Number of subjects)	Median (25-75th percentile)	
Al	CSF (μg/l)	4.2 ± 2.1 (122)	3.7 (2.9-5.2)	4.4 ± 3.1 (50)	3.3 (2.6-5.7)	6.8 ± 3.8 (18)	5.3 (4.4-10)	H test=11.776, df=2, p=0.003, AD vs HC (p=0.003), MCI vs HC (p=0.003)
As	CSF (μg/l)	0.35 ± 0.24 (124)	0.323 (0.256-0.395)	0.307 ± 0.143 (50)	0.289 (0.225-0.376)	0.308 ± 0.095 (19)	0.300 (0.256-0.334)	H test=3.763, df=2, p=0.152
	Plasma (μg/l)	1.159 ± 2.542 (93)	0.730 (0.600-0.970)	1.093 ± 1.426 (35)	0.780 (0.610-1.04)	1.036 ± 1.176 (15)	0.680 (0.500-0.940)	H test=1.615, df=2, p=0.446
B	CSF (μg/l)	29.7 ± 17.7 (124)	27.4 (16.2-41.9)	34.9 ± 43.6 (50)	28.2 (15-37.6)	23.7 ± 18.5 (19)	21.1 (11-30.6)	H test=3.056, df=2, p=0.217
	Plasma (μg/l)	30.5 ± 13.7 (93)	27.5 (21.6-34.6)	37.5 ± 34.4 (35)	26.5 (20.7-45.9)	22.4 ± 8.2 (15)	22.5 (14.5-28.4)	H test=5.387, df=2, p=0.068
Ba	CSF (μg/l)	40.6 ± 24.2 (123)	36.5 (22.6-55.3)	36.9 ± 20.8 (50)	33.4 (21.6-51)	30.5 ± 28.9 (18)	33 (0.4-55.1)	H test=2.696, df=2, p=0.260
Ca	CSF (mg/l)	44.8 ± 14.1 (124)	44.2 (34-55)	40.3 ± 11.7 (50)	37.9 (32-47.7)	40.5 ± 13.1 (19)	39 (31.5-49.7)	H test=4.553, df=2, p=0.103
	Plasma (mg/l)	77.7 ± 7.1 (93)	78.1 (73.5-82.5)	79.5 ± 8.1 (35)	78.3 (74.1-81.2)	81.1 ± 4.4 (15)	82.4 (75.6-85.7)	H test=5.284, df=2, p=0.071
Cd	CSF (μg/l)	0.023 ± 0.012 (124)	0.021 (0.015-0.029)	0.024 ± 0.016 (50)	0.021 (0.013-0.031)	0.022 ± 0.008 (19)	0.021 (0.015-0.027)	H test=0.024, df=2, p=0.988

	Plasma (μg/l)	0.032 ± 0.026 (93)	27.1 (21.6-34.9)	0.034 ± 0.013 (35)	0.032 (0.025-0.041)	0.030 ± 0.021 (15)	0.025 (0.014-0.038)	H test=5.379, df=2, p=0.068
Co	CSF (μg/l)	0.142 ± 0.073 (124)	0.122 (0.091-0.178)	0.151 ± 0.142 (50)	0.115 (0.081-0.174)	0.122 ± 0.081 (18)	0.099 (0.068-0.151)	H test=2.895, df=2, p=0.235
	Plasma (μg/l)	0.433 ± 0.117 (93)	0.430 (0.355-0.49)	0.454 ± 0.153 (35)	0.430 (0.360-0.52)	0.371 ± 0.143 (15)	0.390 (0.240-0.460)	H test=3.564, df=2, p=0.168
Cu	CSF (μg/l)	16.8 ± 6.4 (124)	16.4 (12-20.3)	15.4 ± 6.3 (50)	14.4 (10.8-19.6)	15.3 ± 8.4 (19)	13.1 (8.5-20.4)	H test=2.388, df=2, p=0.303
	Plasma (μg/l)	913.8 ± 190.2 (93)	910 (789.5-1038.5)	966.1 ± 212.1 (35)	969 (842-1089)	1019.2 ± 254.4 (15)	998 (869-1206)	H test=3.319, df=2, p=0.190
Fe	CSF (μg/l)	36.7 ± 20.7 (124)	33.3 (21.5-47.4)	35.2 ± 18.2 (50)	29.9 (22-44.4)	31 ± 13 (19)	30.8 (20.6-39.9)	H test=0.529, df=2, p=0.768
	Plasma (μg/l)	1128 ± 460.5 (79)	996 (849-1283)	1198.5 ± 654.4 (33)	1136 (837.5-1354.5)	883.4 ± 424.1 (11)	838 (498-1100)	H test=3.73, df=2, p=0.155
Hg	CSF (μg/l)	0.066 ± 0.097 (124)	0.051 (0.036-0.077)	0.054 ± 0.037 (50)	0.045 (0.035-0.075)	0.044 ± 0.032 (19)	0.039 (0.024-0.053)	H test=4.492, df=2, p=0.106
	Plasma (μg/l)	0.398 ± 0.518 (93)	0.214 (0.092-0.422)	0.339 ± 0.374 (35)	0.178 (0.133-0.424)	0.332 ± 0.576 (15)	0.102 (0.034-0.377)	H test=2.960, df=2, p=0.228
K	CSF (mg/l)	129.3 ± 38.3 (124)	128.2 (103.2-151.4)	117.7 ± 36.9 (50)	116.5 (90-146.1)	121.8 ± 40.8 (19)	110.7 (100.6-134.3)	H test=4.031, df=2, p=0.133
Li	CSF (μg/l)	2.6 ± 5.4 (124)	1.3 (0.2-2.9)	2.2 ± 3.4 (50)	0.8 (0.2-2.8)	2.8 ± 4.5 (19)	1.7 (0.2-4.0)	H test=0.436, df=2, p=0.804
	Plasma (μg/l)	43.3 ± 34.2 (93)	27 (19-61)	52.7 ± 33 (35)	41 (25-75)	29 ± 13.4 (15)	26 (16-40)	H test=7.101, df=2, p=0.029, AD vs MCI (p=0.045)

Mg	CSF (mg/l)	30.2 ± 8.5 (124)	29.9 (24.5-35.9)	27.9 ± 8.6 (50)	27.8 (21.6-34.2)	27.8 ± 8.3 (19)	24.8 (23.5-30.1)	H test=3.51, df=2, p=0.173
	Plasma (mg/l)	24.2 ± 2.7 (93)	24.3 (22.6-25.7)	24.8 ± 3.4 (35)	23.8 (22.6-25.7)	24.3 ± 6.1 (15)	22.8 (20.6-26.5)	H test=1.104, df=2, p=0.576
Mn	CSF (µg/l)	1.330 ± 0.793 (124)	1.215 (0.762-1.601)	1.215 ± 0.765 (50)	1.044 (0.647-1.487)	1.240 ± 0.841 (19)	1.030 (0.685-1.382)	H test=1.869, df=2, p=0.393
	Plasma (µg/l)	1.175 ± 0.463 (93)	1.080 (0.940-1.245)	1.098 ± 0.263 (35)	1.030 (0.950-1.24)	1.128 ± 0.645 (15)	1.020 (0.750-1.28)	H test=0.839, df=2, p=0.657
Mo	CSF (µg/l)	0.751 ± 0.514 (124)	0.612 (0.418-0.883)	0.671 ± 0.426 (50)	0.594 (0.387-0.891)	0.716 ± 0.834 (19)	0.468 (0.202-0.656)	H test=2.747, df=2, p=0.253
	Plasma (µg/l)	1.175 ± 0.463 (93)	1.080 (0.940-1.245)	1.098 ± 0.263 (35)	1.030 (0.950-1.24)	1.128 ± 0.845 (15)	1.020 (0.750-1.28)	H test=3.695, df=2, p=0.158
Na	CSF (mg/l)	3528.3 ± 885.5 (124)	3546.7 (2986.6-4268.3)	3200.6 ± 982 (50)	3107.9 (2540.7-3927)	3231.8 ± 708.6 (19)	3163.9 (2815.5-3636.8)	H test=6.684, df=2, p=0.035
	Plasma (mg/l)	3649 ± 312.1 (93)	3689 (3537.5-3857)	3661 ± 305.9 (35)	3649 (3479-3821)	3387.9 ± 345.5 (14)	3457.5 (3145–3644.5)	H test=10.567, df=2, p=0.005, AD vs HC (p=0.004)
Ni	CSF (µg/l)	1.261 ± 0.744 (122)	1.117 (0.727-1.668)	1.393 ± 1.233 (50)	1.017 (0.768-1.685)	1.459 ± 0.817 (16)	1.219 (0.917-2.06)	H test=1.032, df=2, p=0.597
	Plasma (µg/l)	1.723 ± 0.601 (93)	1.620 (1.415-1.935)	1.502 ± 0.416 (35)	1.440 (1.200-1.74)	1.881 ± 0.699 (15)	1.830 (1.530-1.97)	H test=6.458, df=2, p=0.04
P	CSF (mg/l)	14.2 ± 4.1 (61)	14.3 (11-17.5)	14.5 ± 3.6 (19)	14 (11.3-16.9)	10.7 ± 2.1 (9)	10.9 (8.8-12.6)	H test=7.067, df=2, p=0.029, AD vs HC (p=0.029)

	Plasma (mg/l)	110 ± 25.6 (93)	105.2 (95.1-122.2)	106.1 ± 22.9 (35)	105.2 (88.6-119.9)	115.7 ± 35.1 (15)	106.7 (99.4-130.7)	H test=0.804, df=2, p=0.669
Pb	CSF (µg/l)	2.408 ± 2.912 (124)	0.894 (0.238-3.904)	2.283 ± 2.98 (50)	1.016 (0.382-2.94)	2.420 ± 2.731 (19)	1.586 (0.351-3.814)	H test=0.264, df=2, p=0.876
	Plasma (µg/l)	0.370 ± 0.423 (93)	0.249 (0.197-0.381)	0.408 ± 0.421 (35)	0.278 (0.179-0.475)	0.292 ± 0.228 (15)	0.243 (0.092-0.383)	H test=1.342, df=2, p=0.511
S	CSF (mg/l)	17.8 ± 7 (124)	17.2 (12.7-22.4)	15.2 ± 7.5 (50)	14 (9.5-18.2)	15.2 ± 8 (19)	13.4 (10.1-17.8)	H test=8.032, df=2, p=0.018, AD vs MCI (p=0.042)
	Plasma (mg/l)	792.9 ± 106.6 (93)	800 (717.5-863)	811.8 ± 113.2 (35)	799 (737-861)	826 ± 62.7 (15)	821 (782-852)	H test=1.934, df=2, p=0.38
Se	CSF (µg/l)	1.578 ± 0.625 (124)	1.525 (1.115-2)	1.458 ± 0.667 (50)	1.309 (1.000-1.895)	1.436 ± 0.863 (19)	1.144 (0.797-2.109)	H test=3.014, df=2, p=0.958
	Plasma (µg/l)	79.1 ± 16.4 (93)	77.7 (69.2-91.3)	86.6 ± 21.3 (35)	83.7 (70.8-96.2)	80.4 ± 17.3 (15)	77.3 (69.3-87.9)	H test=2.449, df=2, p=0.294
Sr	CSF (µg/l)	10.446 ± 7.481 (124)	8.987 (6.378-12.372)	10.035 ± 6.359 (50)	8.550 (6.511-11.95)	9.309 ± 4.091 (19)	7.900 (6.785-11.607)	H test=0.085, df=2, p=0.804
	Plasma (µg/l)	23.8 ± 12.7 (93)	21.4 (18-25.7)	26.9 ± 9 (35)	23.7 (21.1-31)	23.3 ± 5.7 (15)	22.2 (20-25.3)	H test=6.471, df=2, p=0.039
Tl	CSF (µg/l)	0.0057 ± 0.0027 (124)	0.005 (0.004-0.008)	0.0051 ± 0.0029 (50)	0.005 (0.003-0.0073)	0.0061 ± 0.0027 (19)	0.006 (0.004-0.008)	H test=2.693, df=2, p=0.26
	Plasma (µg/l)	0.022 ± 0.008 (93)	0.020 (0.017-0.025)	0.024 ± 0.008 (35)	0.025 (0.018-0.031)	0.024 ± 0.007 (15)	0.024 (0.021-0.029)	H test=5.815, df=2, p=0.055
	CSF	94.2 ± 44.3	88.8	83.4 ± 52.7	67.9	66.8 ± 45.6	47.2	H test=8.96,

Zn	(μg/l)	(124)	(57.6-119.7)	(50)	(48.2-107.1)	(19)	(26.7-97.8)	df=2, p=0.011, AD vs HC (p=0.024)
	Plasma (μg/l)	683.6 ± 100.1 (92)	667.5 (625.3-741)	724.7 ± 116.9 (35)	729 (646-797)	754.7 ± 139.6 (15)	734 (628-828)	H test=5.593, df=2, p=0.061

AD, Alzheimer's disease; CSF, cerebrospinal fluid; HC, healthy controls; MCI, mild cognitive impairment subjects; SD, standard deviation. *$p \leq 0.05$.
